# Molecular profiles of screen detected vs. symptomatic breast cancer and their impact on survival: results from a clinical series

**DOI:** 10.1186/1471-2407-13-15

**Published:** 2013-01-10

**Authors:** Anna Crispo, Maddalena Barba, Giuseppe D’Aiuto, Michelino De Laurentiis, Maria Grimaldi, Massimo Rinaldo, Giuseppina Caolo, Massimiliano D’Aiuto, Immacolata Capasso, Emanuela Esposito, Alfonso Amore, Maurizio Di Bonito, Gerardo Botti, Maurizio Montella

**Affiliations:** 1 Epidemiology Unit, National Cancer Institute G. Pascale Foundation, Via Mariano Semmola, Naples 80131, Italy; 2 Scientific Direction-Division of Medical Oncology B, Regina Elena National Cancer Institute, Via Elio Chianesi 53, Rome 00144, Italy; 3 Breast Unit, National Cancer Institute G. Pascale Foudation, Via Mariano Semmola, Naples 80131, Italy; 4 Medical Oncology, National Cancer Institute G. Pascale Foundation, Via Mariano Semmola, Naples 80131, Italy; 5 Pathology Unit, National Cancer Institute G Pascale Foundation, Via Mariano Semmola, Naples 80131, Italy

**Keywords:** Breast cancer, Mode of detection, Screening, Molecular categories, Survival outcomes

## Abstract

**Background:**

Stage shift is widely considered a major determinant of the survival benefit conferred by breast cancer screening. However, factors and mechanisms underlying such a prognostic advantage need further clarification. We sought to compare the molecular characteristics of screen detected *vs.* symptomatic breast cancers and assess whether differences in tumour biology might translate into survival benefit.

**Methods:**

In a clinical series of 448 women with operable breast cancer, the Kaplan-Meier method and the log-rank test were used to estimate the likelihood of cancer recurrence and death. The Cox proportional hazard model was used for the multivariate analyses including mode of detection, age at diagnosis, tumour size, and lymph node status. These same models were applied to subgroups defined by molecular subtypes.

**Results:**

Screen detected breast cancers tended to show more favourable clinicopathological features and survival outcomes compared to symptomatic cancers. The luminal A subtype was more common in women with mammography detected tumours than in symptomatic patients (68.5 vs. 59.0%, p=0.04). Data analysis across categories of molecular subtypes revealed significantly longer disease free and overall survival for screen detected cancers with a luminal A subtype only (p=0.01 and 0.02, respectively). For women with a luminal A subtype, the independent prognostic role of mode of detection on recurrence was confirmed in Cox proportional hazard models (p=0.03). An independent role of modality of detection on survival was also suggested (p=0.05).

**Conclusions:**

Molecular subtypes did not substantially explain the differences in survival outcomes between screened and symptomatic patients. However, our results suggest that molecular profiles might play a role in interpreting such differences at least partially.

Further studies are warranted to reinterpret the efficacy of screening programmes in the light of tumour biology.

## Background

Consistent evidence from randomized controlled trials of mammography in breast cancer screening demonstrates a 20-35% reduction in mortality from the disease [1-3]. On this basis, the Council of Europe recommends population-based organized mammographic screenings for women aged 50–69 years and claims that screening programmes fulfil the European guidelines [1,2].

In Italy, as well as in most European countries, different modalities of breast cancer screening coexist. The Italian Ministry of Health supports the activation and monitoring of organized breast cancer screening programmes. Asymptomatic women in the aforementioned age range are individually identified and invited to attend mammography screenings. Key issues such as eligibility criteria, quality assurance, follow up of positive results and programme evaluation are centrally regulated and comply with national and international guidelines [1,2]. Conversely, in opportunistic screenings, attendance depends on the individuals decision or on the recommendations given by health care providers. The decentralized nature and lack of systematic reports on activities and outcomes represent further distinctive features [3,4].

Independently on whether organized or opportunistic, breast cancer screening seems to impact cancer prognosis. Screening detected breast cancer cases tend to show a more favorable prognosis compared to cancers clinically detected. This has been partly ascribed to differences in tumour characteristics at diagnosis (e.g. tumour stage and grade, axillary lymph node involvement). However, the persistence of a survival benefit after adjusting for such characteristics suggests an independent role of mode of breast cancer detection on patient prognosis [4-9]. Factors and mechanisms underlying such a prognostic advantage have not been fully elucidated yet.

In recent years, microarrays have allowed the identification and characterization of distinct breast cancer subtypes, namely, luminal A, luminal B, HER2 overexpressing and triple negative tumours. The molecular heterogeneity reflects alterations in cell biology and is associated with significant differences in clinical outcomes [8,10].

Immunohistochemical techniques have contributed details to the characterization of breast cancer subtypes. Luminal A and luminal B breast cancers express the oestrogen receptor (ER) and are also frequently progesterone receptor (PR) positive. HER2 expression is described in the HER2-overexpressing and luminal B subtypes, whereas triple-negative breast cancers are defined by lack of ER, PR and ERBB2 amplification [11-13].

We have previously addressed mode of breast cancer detection in relation to diagnostic delay [8]. In the present study, we sought to compare the molecular characteristics of screen detected *vs.* symptomatic breast cancers and to assess whether differences in tumour biology might translate into survival benefit.

## Methods

### Study participants

We conducted the present analysis on data derived from a clinical series of 448 women diagnosed with incident, histologically-confirmed breast cancer at the G. Pascale National Cancer Institute of Naples, between January 2004 and June 2006. Detailed eligibility criteria were reported elsewhere [14]. In brief, breast cancer patients were included if aged ≥18 years and tumour samples were available for molecular and immunohistochemical characterization.

Data on pathologic features (e.g. tumour size and grade at diagnosis), administered therapy, and survival outcomes were gathered from our patient and pathology databases. A validated, semi-structured questionnaire was administered in face-to-face interviews to collect data on demographics and mode of breast cancer detection. Tumours were considered screen detected if suspicious findings were first detected by breast imaging within the routine national screening program or by opportunistic screening without any symptoms. Conversely, in patients with symptomatic tumours, breast imaging was performed in the absence of screening and exclusively following self breast examination or examination by an experienced health care provider revealing symptoms related to breast cancer, e.g. palpable lumps, changes in the skin over the breast, changes in the shape and/or size of the breast.

In asymptomatic women aged 50 years and older, participation in the national screening program was assessed throughout a specifically tailored question on whether they had undergone mammography following an invitation letter from the local authority.

### Immunohistochemistry

Antigen expression was evaluated by an experienced pathologist using light microscopy. The observer was unaware of the clinical outcome. For each sample, at least five fields (inside the tumour and in the area exhibiting tumour invasion) and >500 cells were analyzed. Using a semiquantitative scoring system, the intensity, extent and subcellular distribution of ER, PR, c-erb B2, Ki67, CK 5/6, CK 14 and CK8/18 were evaluated.

The cutoff used to distinguish “positive” from “negative” cases was ≥ 1% ER/PR positive tumour cells. Immunohistochemical analyses of HER2 expression describe the intensity and staining pattern of tumour cells. Only membrane staining intensity and pattern were evaluated using the 0 to 3+ score as illustrated in the HercepTest kit scoring guidelines. The FDA-recognized test, the Herceptest™ (DAKO), describes four categories: no staining, or weak staining in fewer than 10% of the tumour cells (0); weak staining in part of the membrane in more than 10% of the tumour cells (1+); complete staining of the membrane with weak or moderate intensity in more than 10% of the neoplastic cells (2+); and strong staining in more than 10% (3+). Scores of 0 or 1+ were considered negative for HER2 expression, 2+ was uncertain, and 3+ was positive. Cases 2 + undergo FISH analysis.

The proliferative index Ki67 was defined as the percentage of immunoreactive tumour cells out of the total number of cells. The percentage of positive cells per case was scored according to 2 different groups: group 1: <15% (low proliferative activity); group 2: >15% (high proliferative activity).

CKs stains were considered positive if any (weak or strong) cytoplasmic and/or membranous invasive carcinoma cell staining was observed.

### Molecular subtype classification

Breast cancers were classified into five molecular subtypes based on the expression of ER, PR, HER2, and basal cytokeratins as follows: luminal A tumours (ER+ or PR+, and HER2-), luminal B tumours (ER+ or PR+, and HER2+), non-luminal HER2+ tumours (ER-, PR-, and HER2+), triple negative with expression of core basal markers (ER-, PR-, HER2-, and CK5/6+ and/or CK14+ and CK8/18-) and triple negative without expression of core basal markers (ER-, PR-, HER2-, and CK5/6- and/or CK14- and CK8/18**+**).

### Statistical analyses

Frequency tables were analyzed using the Chi-Square test. The date of last follow-up for relapse-free or living patients was 31-12-2010. Time from diagnosis to relapse was recorded; time from diagnosis to development of metastatic disease or death was then calculated and survival was compared by mode of cancer detection.

Estimation of the likelihood events for locoregional, distant failure and overall survival (OS) were calculated according to the Kaplan-Meier method. Statistical differences between curves were calculated using log-rank test [15,16].

The Cox proportional hazard model was used to test the effect of several variables on survival outcomes in multivariate analyses [17]. Mode of breast cancer detection, age at cancer diagnosis, tumour size, number of positive lymph nodes and, when analyzing the overall sample, molecular subtypes were included as covariates. The same models were applied to subgroups defined by molecular subtypes. In addition, regression models were used to test the interaction between mode of breast cancer detection and each molecular subtype. A *p* value of <0.05 was considered significant. Statistical analysis was performed using SPSS (version 16; SPSS, Inc., Chicago, IL).

## Results

In Table 1, patient characteristics are reported by mode of breast cancer detection. Of these women, 334 (74.5%) had symptomatic tumours and 114 (25.5%) had screen detected tumours. In the screen detected group, only three women among those aged 50 years and older (3.61%) reported having undergone a mammography after receiving an invitation letter from the local health authority. Median follow-up for the overall sample was 61.8 months (range 4–83 months). Women who were symptomatic at diagnosis were more commonly younger, with the proportion of patients aged ≤ 49 years being significantly higher compared to women in the mammography group (37.5% vs. 27.2%, p<0.0001). Patients with a symptomatic cancer were also more frequent in unmarried cases (28.9% vs. 15.9%, p=0.006).

**Table 1:** Descriptive characteristics of the study participants by mode of breast cancer detection

	**MODE OF DETECTION**				
**Descriptive Characteristics**	**SYMPTOMATIC**		**SCREEN DETECTED**		**P-VALUE**
	**No.**	**%**	**No.**	**%**	
TOTAL	334	74.5	114	25.5	
**Age at diagnosis (years)**					<.0001
<40	47	14.1	3	2.6	
40–49	78	23.4	28	24.6	
50–69	150	44.9	77	67.5	
≥70	59	17.7	6	5.3	
**Education (years)**					.06
<7	144	43.9	36	31.6	
7–11	74	22.6	30	26.3	
≥12	110	33.5	48	42.1	
**Marital status**					.006
Married/living as married	236	71.1	95	84.1	
Not married	96	28.9	18	15.9	
**Menopausal status**					.06
Premenopausal	93	27.8	20	17.5	
Perimenopausal	19	5.7	10	8.8	
Postmenopausal	222	66.5	84	73.7	
**Body mass index**					.24
<25	132	41.4	48	44.9	
25–29	105	32.9	40	37.4	
≥30	82	25.7	19	17.8	
**Family History**					.51
None	232	69.5	73	64.0	
FDR (≥1)	54	16.2	18	15.8	
SDR (≥1)	41	12.3	19	16.7	
FDR+SDR	7	2.1	4	3.5	

Abbreviations: BC, breast cancer; FDR, first-degree relative; SDR, second-degree relative.

Table 2 summarizes the baseline clinical, pathological and immunohistochemical characteristics, along with the administered treatment and outcomes of interest by mode of breast cancer detection. Screen detected cancers were smaller, more likely to be node-negative, and better differentiated than symptomatic ones (80.8 vs. 51.7%, p<0.0001; 70.2 vs. 52.8%, p=0.01 and 18.5 vs. 7.2%, p<0.0001, respectively).

**Table 2:** Tumor characteristics, type of surgery, adjuvant treatment and survival outcomes by mode of breast cancer detection

	**MODE OF DETECTION**				
	**SYMPTOMATIC**		**SCREEN DETECTED**		**P-VALUE**
**TUMOR CHARACTERISTICS**	**No.**	**%**	**No.**	**%**	
TOTAL	334	74.5	114	25.5	
**Tumor size**					<.0001
T1	166	51.7	84	80.8	
T2	123	38.3	19	18.3	
T3–T4	32	10.0	1	1.0	
**No. of positive Lymph nodes**					.01
All lymph nodes negative	167	52.8	73	70.2	
1–3	77	24.4	20	19.2	
4–9	52	16.5	9	8.7	
≥10	20	6.3	2	1.9	
**Histological grade**					<.0001
1	23	7.2	20	18.5	
2	120	37.7	49	45.4	
3	175	55.0	39	36.1	
**Immunoistochemical characteristics**					
**ER status**					.11
Positive	236	70.7	88	77.2	
Negative	98	29.3	26	22.8	
**PR status**					.04
Positive	227	68.0	89	78.1	
Negative	107	32.0	25	21.9	
**Ki-67**					.02
≤20%	141	44.1	60	57.1	
>20%	179	55.9	45	42.9	
**HER2 status**					.96
0, 1+	227	70.1	76	70.4	
2+	46	14.2	16	14.8	
3+	51	15.7	16	14.8	
**Molecular Subtypes**					.04
Luminal A	193	59.0	74	68.5	.09
Luminal B	59	18.2	18	16.7	.71
Non-Luminal HER2+	38	11.0	14	13.0	.79
No Basal-like	18	5.6	2	1.8	.01
Basal-like	16	4.9	0		
**TREATMENT CHARACTERISTICS**					
**Type of surgery**					.005
Conservative	242	74.7	88	88.0	
Mastectomy	82	25.3	12	12.0	
**Adjuvant Therapy**					.04
None	36	11.8	15	13.6	
Radiation only	64	21.1	37	33.6	
Chemotherapy only	33	10.9	8	7.3	
Radiation and Chemotherapy	171	56.2	50	45.5	
**Hormone given**	244	81.3	92	83.6	.23
**OUTCOME CHARACTERISTICS**					
**Vital status**					<.0001
Alive, no evidence of disease	226	69.7	100	88.4	
Alive with this cancer	40	12.3	5	4.4	
Dead, no evidence of disease	4	1.2	0	-	
Dead from this cancer	54	16.6	8	7.0	
**Recurrence type**					.001
None	230	71	100	88.5	
Loco-Regional	18	5.6	3	2.7	
Distant	76	23.5	10	8.8	

Abbreviations: BC, breast cancer; Subtypes: Luminal A (ER+ and/or PR+) and HER2-; Luminal B (ER+ and/or PR+) and HER2+; Non-Luminal HER2+ (ER- and PR-) and HER2+; Triple Negative (ER-, PR- and HER2-).

Abbreviations: BC, breast cancer.

A significantly higher proportion of cases expressed PgR and showed a Ki-67 ≤20% among screen detected cancers compared with symptomatic tumours (78.1% vs. 68%, p=0.04 and 57.1% vs. 44.1%, p=0.02, respectively). Triple negative cancers were more common among the self-detected cases than among the mammography ones (10.2 vs. 1.7%, p= 0.006).

For women in the screen detected category, the surgical approach tended to be more conservative and chemotherapy, either alone or combined to radiotherapy, was less frequently administered compared to women who were symptomatic at diagnosis (88.0 vs. 74.7%, p=0.005; 7.3% vs. 10.9% and 45.5% vs. 56.2%, p=0.04, respectively). A significantly higher percentage of women with symptomatic cancer died from the disease during the follow up compared to patients whose cancer was screen detected (16.6 vs. 7.0%, p<0.0001). Patients alive at the last follow up were more commonly free from the disease if their cancer had been screen detected (88.5% vs. 71.0%, p<0.0001). Loco-regional and distant recurrence were more likely to occur in patients with a symptomatic cancer than in patients with a screen detected cancer (23.5 vs. 8.8% and 5.6 vs. 2.7%, respectively, p<0.001).

In univariate analyses, mode of breast cancer detection was an independent predictor of both recurrence and survival (HR: 2.5, 95% CI 1.4-4.5 and HR: 2.5, 95% CI 1.2-5.4, respectively), with women in the screen detected category showing better outcomes compared to women with symptomatic cancers (p=0.001 and p=0.007 for recurrence and death, respectively). Age at diagnosis, tumour size, nodal status and molecular subtypes were also associated with survival outcomes (data available upon request).

Regression models including exclusively molecular subtypes showed a significant impact of mode of detection on the outcomes of interest (HR 2.26, 95% CI 1.26-4.06; HR 2.24, 95% CI 1.06-4.73, for recurrence and death, respectively). However, this result was not confirmed when further adjusting for age, tumour size and nodal status (HR: 2.02, 95% CI 0.97-4.18 and HR: 2.68, 95% CI 0.92-7.77, for recurrence and death, respectively). Age remained an independent prognostic factor for death only (p=0.001), while nodal status had an impact on both recurrence and death (p=0.0001). Data also showed the molecular subtype role on disease recurrence (p= 0.0001) (Table 3).

**Table 3:** Cox multivariate analysis of disease-free and overall survival

	**Recur HR (95% CI)**	**P-value**	**Death HR (95% CI)**	**P-value**
**Mode of BC Detection**				
Screen Detection	1.00		1.00	
Symptomatic	2.02 (0.97–4.18)	.059	2.68 (0.92–7.77)	.07
**Age**		**.21**		**.001**
<40	1.00		1.00	
40–49	0.72 (0.34–1.54)	.42	1.24 (0.32–4.93)	.71
50–69	1.19 (0.62–2.28)	.63	3.61 (1.09–11.99)	.04
≥70	1.44 (0.67–3.08)	.35	6.56 (1.83–23.47)	.004
**Tumor size**		**.12**		**.14**
T1	1.00		1.00	
T2	1.36 (0.87–2.15)	.23	1.58 (0.86–2.91)	.11
T3–T4	2.03 (1.01–4.06)	.04	2.32 (0.96–5.6)	.06
**No. of positive Lymph nodes**		**<.0001**		**<.0001**
All lymph nodes negative	1.00		1.00	
1–3	2.22 (1.28–3.84)	.004	1.66 (0.77–3.54)	.19
≥4	5.31 (3.15–8.96)	<.0001	6.09 (3.07–11.96)	<.0001
Molecular Subtypes*		**<.0001**		**.22**
Luminal A	1.00		1.00	
Luminal B	1.46 (0.86–2.47)	.21	1.04 (0.51–2.14)	.89
Non-Luminal HER2+	1.80 (0.97–3.35)	.06	2.23 (1.07–4.67)	.03
Non Basal-like	1.75 (0.68–4.53)	.22	1.08 (0.25–4.67)	.91
Basal-like	4.72 (2.13–10.46)	<.0001	2.06 (0.6–7.05)	.24

Abbreviations: BC, breast cancer; D-FS, disease-free survival; OS, overall survival; HR, hazard ratio; * Molecular Subtypes: Luminal A (ER+ and/or PR+) and HER2-; Luminal B (ER+ and/or PR+) and HER2+; Non-Luminal HER2+ (ER- and PR-) and HER2+; Triple Negative (ER-, PR- and HER2-: CK5+ Basal-like; Non Basal-like).

In Table 4, tumour characteristics are reported by molecular subtypes. Among women with a luminal A subtype, screen detected cases were more commonly aged 50 or older, exhibited smaller tumours, and lower histological grade compared with symptomatic patients (74.3 vs. 67.4%, p=0.001; 87.0 vs. 55.4%, p=0.001 and 27.8 vs. 11.5%, p=0.006 for age at diagnosis, T≤2 cm and grade 1, respectively). In the luminal B subcategory, histological grade was significantly lower in screen detected cases than in symptomatic cancers (77.8 vs. 35.6, p=0.002). In the HER2+ subtype, lymph node involvement was less common in screen detected cases than in symptomatic patients (91.7 vs. 38.9, p=0.006).

**Table 4:** Tumor characteristics by molecular subtypes

	**Luminal A**			**Luminal B**			**Non-Luminal HER2+**			**Triple Negative**		
	**N = 267**			**N = 77**			**N = 52**			N = 36*		
	**Symptomatic**	**Screen Detected‡**	***p-value***	**Symptomatic**	**Screen Detected**	***p-value***	**Symptomatic**	**Screen Detected**	***p-value***	**Symptomatic**	**Screen Detected**	***p-value***
	**(N, *%*)**	**(N, *%*)**		**(N, *%*)**	**(N, *%*)**		**(N, *%*)**	**(N, %)**		**(N, *%*)**	**(N, *%*)**	
**Age**			***.001***						***.34***			***.31***
<40 years	24 (12.4)	2 (2.7)		9 (15.3)	0		6 (15.8)	1 (7.1)		4 (11.8)	0	
40–49	39 (20.2)	17 823)		16 (27.1)	2 (11.1)		9 (23.7)	6 (42.9)		11 (32.4)	0	
50–69	93 (48.2)	51 (68.9)		25 (42.4)	14 (77.8)		18 (47.4)	7 (50)		12 (35.3)	2	
≥70	37 (19.2)	4 (5.4)		9 (15.3)	2 (11.1)		5 (13.2)	0		7 (20.6)	0	
**Tumor size**			***<.0001***			***.53***			***.11***			***.14***
T1	103 (55.4)	60 (87)		32 (55.2)	11 (64.7)		16 (44.4)	9 (75)		10 (30.3)	2 (100)	
T2	60 (32.3)	9 (13)		22 (37.9)	6 (35.3)		17 (47.2)	2 (16.7)		22 (66.7)	0	
T3–T4	23 (12.4)	0		4 (6.9)	0		3 (8.3)	1 (8.3)		1 (3.0)	0	
**Lymph nodes**			***.21***			***.22***			***0.06***			***.31***
None	101 (54.3)	45 (66.2)		27 (47.4)	12 (66.7)		14 (38.9)	11 (91.7)		22 (64.7)	1 (50)	
1–3	41 (22)	13 (19.1)		19 (33.3)	5 (27.8)		14 (38.9)	1 (8.3)		4 (11.8)	1 (50)	
≥4	44 (23.7)	10 (14.7)		11 (19.3)	1 (5.6)		8 (22.2)	0		8 (23.5)	0	
**Histological grade**			***.006***			***.002***			***.81***			***.81***
1	21 (11.5)	20 (27.8)					1 (2.7)	0				
2	88 (48.1)	29 (40.3)		21 (35.6)	14 (77.8)		8 (21.6)	3 (23.1)		1 (3)	0	
3	74 (40.4)	23 (31.9)		38 (64.4)	4 (22.2)		28 (75.7)	10 (76.9)		32 (97)	2 (100)	
**Ki-67**			***.15***			***.11***			***.23***			***.72***
≤20%	102 (54.8)	46 (64.8)		22 (37.3)	10 (55.6)		12 (33.3)	2 (16.7)		2 (6.1)	0	
>20%	84 (45.2)	25 (35.2)		37 (62.7)	8 (44.4)		24 (66.7)	10 (83.3)		31 (93.9)	2 (100)	

‡ MG: Mammography; *36 TN: 20 Non Basal-like, 16 Basal-like.

**Figure 1:**
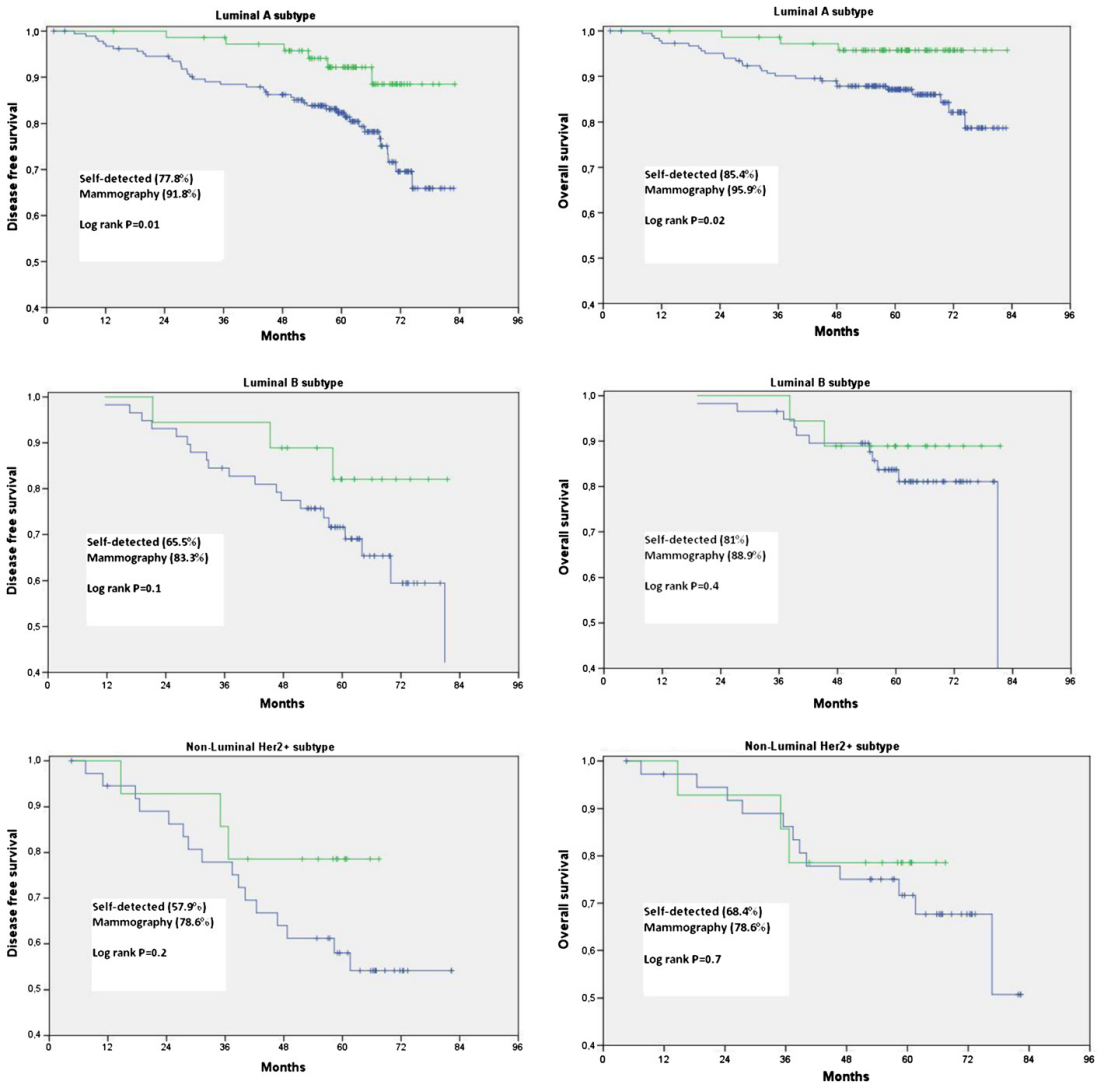
**Effect of method of detection on disease-free and overall survival according to the molecular subtypes.** A disease-free and B overall survival for luminal A subtype. C disease-free and D overall survival for luminal B subtype. E disease-free and F overall survival for HER2+ subtype.

In Figure 1, survival outcomes are shown by molecular subtypes. In the luminal A subgroup, screen detected cancers had significantly better outcomes than symptomatic patients [91.8 vs. 77.8%, log rank=0.01 and 95.9 vs. 85.4%, log rank=0.02 for disease free survival (DFS) and overall survival (OS), respectively].

We then tested variables to identify predictors of survival outcomes by applying Cox proportional hazard models within strata of molecular subtypes (Table 5). In the luminal A subtype, the multivariate analysis including tumour size and nodal status confirmed the role of mode of breast cancer detection in affecting cancer recurrence (p=0.03), while estimates on survival were of borderline significance (p=0.05). In this same subset of patients, lymph node involvement significantly affected both recurrence and death (p≤0.0001). In the HER2+ subtype, tumour size was an independent predictor of recurrence (p=0.03). In triple negative cancers, tumour size showed a significant impact on recurrence (p=0.03), while nodal status influenced both recurrence and death (p=0.001 and p=0.01, respectively).

**Table 5:** Cox multivariate analysis of disease-free and overall survival by molecular subtypes

	**Recur**	**P-value**	**Death**	**P-value**
	**HR (95% CI)**		**HR (95% CI)**	
***Luminal A***				
**Mode of BC Detection**				
Screen Detected	1.00		1.00	
Symptomatic	2.73 (1.05–7.13)	.03	4.2 (0.97–18.16)	.05
**Tumor size**		**.91**		**.72**
T1	1.00		1.00	
T2	1.11 (0.56–2.2)	.73	1.19 (0.51–2.78)	.74
T3–T4	1.09 (0.42–2.87)	.81	0.69 (0.18–2.58)	.52
**No. of positive Lymph nodes**		**<.0001**		**<.0001**
All lymph nodes negative	1.00		1.00	
1–3	1.9 (0.81–4.47)	.12	2.84 (0.89–9.05)	.07
≥4	6.27 (3.05–12.91)	<.0001	9.69 (3.57–26.26)	<.0001
***Luminal B***				
**Mode of BC Detection**				
Screen Detected	1.00		1.00	
Symptomatic	2.66 (0.59–11.88)	.21	2.79 (0.33–23.7)	.33
**Tumor size**		**.4**		**.05**
T1	1.00		1.00	
T2	1.05 (0.39–2.77)	.91	0.56 (0.11–2.92)	.53
T3–T4	2.61 (0.58–11.71)	.22	5.59 (1.03–30.22)	.04
**No. of positive Lymph nodes**		**.12**		**.74**
All lymph nodes negative	1.00		1.00	
1–3	2.55 (0.82–7.88)	.01	0.81 (0.14–4.52)	.81
≥4	3.55 (1.02–12.42)	.04	1.66 (0.35–7.75)	.52
***Non Luminal Her2+***				
**Mode of BC Detection**				
Screen Detected	1.00		1.00	
Symptomatic	1.86 (0.19–18.18)	.72	1.43 (0.14–14.05)	.91
**Tumor size**		**.03**		**.09**
T1	1.00		1.00	
T2	1.77 (0.47–6.67)	.44	5.48 (1.02–29.2)	.05
T3–T4	8.62 (1.65–44.78)	.01	8.56 (1.05–69.9)	.04
**No. of positive Lymph nodes**		**.81**		**.42**
All lymph nodes negative	1.00		1.00	
1–3	1.47 (0.38–5.6)	.84	0.41 (0.09–1.88)	.22
≥4	1.54 (0.34–7.05)	.51	1.07 (0.22–5.1)	.91
***Triple Negative***				
**Mode of BC Detection**				
				
Screen Detected	1.00		1.00	
Symptomatic	0.14 (0.008–2.39)	.11	n.e*.	
**Tumor size**		**.11**		**.12**
T1	1.00		1.00	
≥T2	7.17 (1.15–44.72)	.03	7.41 (0.61–89.8)	.14
**No. of positive Lymph nodes**		**.001**		**.01**
All lymph nodes negative	1.00		1.00	
All lymph nodes positive	3.58 (3.65–7.76)	.001	4.85 (1.46–16.13)	.01

*n.e. not evaluable.

Regression models showed no significant interaction between mode of breast cancer detection and molecular subtype for both the outcomes of interest (available upon request).

## Discussion

In the present study, we analyzed data from a clinical series of 448 women with operable breast cancer and compared characteristics related to patients tumour, treatment, and outcomes by mode of breast cancer detection. We observed more favourable prognostic factors and survival outcomes in women with screen detected breast cancers compared with symptomatic patients. The independent role of mode of breast cancer detection was not confirmed in multivariate analyses including age, tumour size and nodal status. However, adjusted and unadjusted HR did not dramatically differ and the two 95% CIs largely overlapped.

Based on the hypothesis of a potential role of breast cancer biology in explaining the impact of mode of breast cancer detection on survival outcomes, we re- analyzed data within strata defined by molecular subtypes. Overall, screen detected cancers tended to show more favourable prognostic features across the various molecular categories. However, screen detected cancers showed significantly better disease free and overall survival compared to symptomatic tumours in the luminal A subtype only. In this subcategory, the multivariate analyses confirmed the independent role of mode of detection on recurrence, while there was only a suggestion for its role on death.

Breast cancers in the screen detected group tended to be smaller, more often node negative and with lower histological grade compared with tumours in the symptomatic group. Patients in this group tended to be older. Age at breast cancer diagnosis is an independent prognostic factor, with younger age associated to having more aggressive tumour behaviour [18]. Screen detected tumours were more likely to express ER and/or PgR and to show a lower Ki67 index. The more favourable clinicopathological characteristics provide a rationale for the more conservative surgical approach and less frequent administration of adjuvant therapy in screen detected tumours. These findings are consistent with the results of previous studies [6,7].

In our study, screen detection was associated with better survival outcomes. The survival advantage conferred by screening has been mostly attributed to stage shift, i.e. the proportional shift toward earlier-stage cancer at presentation. The latter is a reflection of screening-related lead-time bias, which lengthens survival duration and explains, at least partly, the observed improvement in outcomes of patients with screen detected tumours [19-22]. However, consistent evidence supports an independent prognostic role of screen detection after adjustment for disease stage. Indeed, Dawson and colleagues compared the effects of screen detection with symptomatic diagnosis on survival after adjustment for the Nottinghan Prognostic Index, a prognostic indicator based on tumour size, grade and lymph node status. The authors concluded that the shift in NPI accounted only for the fifty-six per cent of the survival benefit associated with screen detection [23].

In our study, symptomatic breast cancer patients tended to be significantly younger. Younger age at breast cancer diagnosis is associated with more aggressive tumour behavior and might help interpret differences in outcomes by mode of detection [24].

Based on pre-set inclusion criteria, inoperable breast cancer cases were excluded from our analysis. We cannot estimate their exact proportion and distribution across the study groups. However, given the tendency of screen detected tumours to show smaller size compared to symptomatic cases, we may presume that inoperable cancer were more represented among symptomatic patients, thus eventually contributing to worse survival outcomes in this subgroup.

Our results might help explain the role of tumour biology in affecting differences in survival outcomes between women diagnosed with screen detected tumours and symptomatic patients. Indeed, we observed a significantly higher percentage of luminal A subtype among women within the screen detected group compared to patients with symptomatic tumours. Conversely, the triple negative subtype was significantly more common among symptomatic patients compared to women with screen detected tumours. This is consistent with the results reported by Kim et al. and Dawson et [23,25]. Molecular subtypes are largely and consistently recognized as predictors of recurrence and death [10-12]. Since the luminal A subtype is usually associated with more favorable outcomes, it is plausible that the significantly higher proportion of this molecular subtype within the screen detected group (compared with the symptomatic group) might explain at least in part the survival advantage observed in women with a screen detected cancer [26-28].

When comparing tumour characteristics and outcomes of interests between screen detected and symptomatic cancers across categories defined on the basis of the molecular subtypes, the number of predictors of a more favorable prognosis was remarkable in the luminal A subtype only. This was the only subtype associated with a significant advantage in survival outcomes. These findings might strengthen the evidence in supporting a selective influence of early detection on survival in less aggres-sive tumours, i.e. luminal A subtype. Conversely, doubts remain concerning the efficacy in the amelioration of survival outcomes for more aggressive tumours.

A limited number of studies have investigated the association of interest so far. Kim and coauthors retrospectively reviewed the clinical and pathologic data from 3,141 patients who underwent surgery for the treatment of invasive breast cancer at the Samsung Medical Center. Consistently with our results, the authors observed more favorable prognostic survival outcomes in screened-detected breast cancers compared with symptomatic cases (5-year OS: 99.7 vs. 96.5%, p=0.001 and DFS: 96.4 vs. 90.7, p<0.001). Screen detection was independently associated with improved OS and DFS after adjustment for covariates (HR=0.32, p=0.0035; HR: 0.58, p=0.020, respectively) [25]. We have previously mentioned the analysis from Dawson et al., including data from 1379 women with invasive breast cancers. The authors identified distinct differences in the molecular characteristics of screen-detected vs. symptomatic breast cancers. However, only minimal attenuation of the screen-detected survival advantage was observed after adjustment for the expression of individual molecular biomarkers or molecular subtype in multivariate analysis. Indeed, the percentage of survival benefit attributable to these factors was 3-10%, with more than 30% of the effect remaining unexplained [23]. In a recent study by Shito and coauthors, screen detection was an independent predictor of favourable distant disease-free survival in multivariate analysis including age, grade and tumour size. According to the authors’ conclusions, differences in molecular subtypes of screen-detected vs. symptomatic breast cancers accounted in part for the better outcome of screen-detected cancers. However, the effect of molecular subtype on the survival advantage conferred by screen detection was not assessed in this analysis [29].

Our study has some limitations. We analyzed data from a clinical series of 448 women with operable breast cancer. The sample size limitations mostly reflect on the non-Luminal A subgroups, which are particularly under-represented among patients included in our analysis. Our study might lack sufficient power to highlight the impact of molecular determinants on survival outcomes by detection mode in non-Luminal A patients*.* When assessing the interaction between mode of breast cancer and molecular subtypes for the outcomes of interest, we observed non significant results. However, interaction effects are often undetectable in subgroup analyses when sufficient power is lacking [30]. The relatively limited sample size and study design, i.e., clinical series, both concur to limit the ability to make definitive interpretation of this data and encourage conducting further research based on specifically conceived, adequately powered, prospective studies.

Mode of breast cancer detection was defined on the basis of self reported data. The remarkably low percentage of women having undertaken mammography within an organized screening program discouraged us from relying on official records to confirm our data. Under these circumstances, misclassification bias cannot be excluded. However, evidence from a validation study of self reported screening mammography histories suggests that non differential rather than differential is a more likely type of error and that the related estimates might understate the effects of screening detection regarding breast cancer outcomes [31].

Our study also has several strengths. Data on demographics and mode of breast cancer detection were collected using a specifically conceived questionnaire which was administered during face-to face interviews. Abstraction of medical records on (breast cancer) pathologic features, treatment and outcomes was carried out by a specifically trained medical assistant who worked in close collaboration with the oncologists who had prospectively followed the patients included in our analyses. This increases our confidence in the quality of the data collected.

In our analyses, we included data concerning a wide panel of molecular biomarkers. In particular, we were able to gather data on biomarkers such as Ki-67, CK 5/6, CK14 and EGFR which were not available in previous studies [25].

## Conclusions

In conclusions, breast cancer patients with mammography detected tumours tended to show more favourable clinicopathological features and survival outcomes compared to women who were symptomatic at cancer diagnosis. Patients with screen detected breast cancers were more likely to exhibit a luminal A subtype.

This is associated with better survival outcomes and might per se explain at least a proportion of the advantage in survival observed in mammography detected cancers. Data analysis across categories of molecular subtypes revealed significantly longer disease free and overall survival for screen detected cancers with a luminal A subtype only. In the luminal A subtype, the independent prognostic role of mode of breast cancer detection on cancer recurrence was confirmed in Cox proportional hazard models. These same models also suggested an independent prognostic role of modality of detection on survival.

Overall, molecular subtypes did not substantially explain differences in survival outcomes between screened and symptomatic patients. However, our results suggest that molecular profiles might play a role in interpreting such differences at least partially. If this is confirmed, the efficacy of screening programmes would be revisited in light of tumour biology.

## Competing interests

There is no conflict of interest: no financial and personal relationships with other people or organizations that could inappropriately influence (bias) the work.

## Authors’ contributions

AC participated in the design of the study, performed statistical analysis and helped to draft the manuscript. MB participated in the design of the study, helped to perform statistical analysis and drafted the manuscript. GD, MDM, MG, MR, GC, MD, IC, EE, AA, MDB and GB participated in the design of the study and revised the manuscript critically for important intellectual content. MM conceived and coordinated the study. All the authors read and approved the final manuscript.

## Pre-publication history

The pre-publication history for this paper can be accessed here:

http://www.biomedcentral.com/1471-2407/13/15/prepub
